# Hypercalcemia Causes More Severe Acute Pancreatitis: An International Multicenter Cohort Study

**DOI:** 10.3390/jcm14176304

**Published:** 2025-09-06

**Authors:** Gábor Gieszinger, Balázs Kui, Péter Hegyi, Péter Jenő Hegyi, Áron Vincze, Bálint Erőss, Andrea Szentesi, Vivien Vass, Zsolt Abonyi-Tóth, Ferenc Izbéki, Anita Illés, Imre Szabó, József Czimmer, Balázs Csaba Németh, László Gajdán, Mária Papp, József Hamvas, László Czakó

**Affiliations:** 1 Department of Internal Medicine, Center for Gastroenterology, Albert Szent-Györgyi Medical School, University of Szeged, 6725 Szeged, Hungary; gieszingergabor@gmail.com (G.G.); k.kubali@gmail.com (B.K.); nemeth.balazs@med.u-szeged.hu (B.C.N.); 2 Centre for Translational Medicine, Semmelweis University, 1094 Budapest, Hungary; hegyi2009@gmail.com (P.H.); drdunajskastreda@gmail.com (P.J.H.); dr.eross.balint@gmail.com (B.E.); abonyi-toth.zsolt@univet.hu (Z.A.-T.); 3 Institute of Pancreatic Diseases, Semmelweis University, 1083 Budapest, Hungary; 4 Institute for Translational Medicine, Medical School, University of Pécs, 7624 Pécs, Hungary; szentesiai@gmail.com (A.S.); vass.vivien@pte.hu (V.V.); 5 Translational Pancreatology Research Group, Interdisciplinary Centre of Excellence for Research, Development and Innovation, University of Szeged, 6725 Szeged, Hungary; 6 Division of Gastroenterology, First Department of Medicine, Medical School, University of Pécs, 7624 Pécs, Hungary; vincze.aron@pte.hu (Á.V.); illes.anita@pte.hu (A.I.); szabo.imre@pte.hu (I.S.); czimmer.jozsef@pte.hu (J.C.); 7 Department of Biostatistics, University of Veterinary Medicine, 1078 Budapest, Hungary; 8 Szent György Teaching Hospital of County Fejér, 8000 Székesfehérvár, Hungary; fizbeki@gmail.com (F.I.); lgajdan@yahoo.com (L.G.); 9 Hungarian Centre of Excellence for Molecular Medicine—University of Szeged, Translational Pancreatology Research Group, University of Szeged, 6728 Szeged, Hungary; 10 Division of Gastroenterology, Department of Internal Medicine, Faculty of Medicine, University of Debrecen, 4032 Debrecen, Hungary; papp.maria@med.unideb.hu; 11 Péterfy Hospital, 1076 Budapest, Hungary; jozsef.hamvas@gmail.com

**Keywords:** hypercalcemia, acute pancreatitis, organ failure, renal failure, heart failure, respiratory failure

## Abstract

**Background**: Hypercalcemia is a rare etiology of acute pancreatitis; only a few cases have been reported in the literature, and the severity of hypercalcemia-induced AP is unknown. We aimed to assess the occurrence and severity of hypercalcemia-induced AP and compare it with the clinical characteristics of AP caused by other etiological factors. **Methods**: We collected data from patients from the Hungarian Acute Pancreatitis Registry who had AP, a serum calcium level above 2.6 mmol/L, and no other AP etiology. AP patients with etiologies other than hypercalcemia served as control. **Results**: A total of 1.20% of our AP patients (16/1328) had a clear hypercalcemic etiology, 5.05% (67/1328) had a mixed etiology, and 1245 patients were in the control group. Severe AP, organ failure, and renal failure were significantly more common in patients with hypercalcemia-induced AP than in the control or the mixed etiology groups. Heart failure was significantly more frequent in the clear hypercalcemia-induced group than in patients with normal serum calcium AP. Respiratory failure was significantly more common in the clear hypercalcemia-induced AP group than in the mixed etiology-induced group. There was no significant difference in other analyzed parameters. The outcome of AP was not associated with the severity of hypercalcemia within the hypercalcemic group. **Conclusions**: Compared with AP of different etiologies, hypercalcemia-induced AP is more likely to develop into severe AP and organ failure (heart and kidneys).

## 1. Introduction

Acute pancreatitis (AP) is one of the most common gastroenterological diseases to require hospital admission. The main etiological factors of AP are gallstones and excessive alcohol consumption, but rarer causes, such as hypercalcemia, should be kept in mind. According to the literature, hypercalcemia causes 0.5% of all AP cases [1,2]. Only a few cases have been reported in the literature, and the severity of hypercalcemia-induced AP is unknown. The most frequent causes of hypercalcemia are endocrine diseases (hormone-producing parathyroid adenomas), lytic bone metastasis, chronic kidney failure, genetic etiology, paraneoplastic syndrome, and drug-induced cases [2]. Calcium is absorbed in the intestines, stored in ionized and non-ionized forms in the blood and bones, and excreted through urine and stool. Parathormone plays a crucial role in maintaining calcium homeostasis. It increases the level of calcium in the blood by transferring calcium from the bones and by absorption from the intestines [3].

The role of calcium in the pathogenesis of AP has been confirmed in numerous publications. Calcium injected into rats increases the serum amylase level, trypsinogen activation, and the level of morphological changes associated with inflammation [4]. It has been demonstrated that intracellular calcium overload and mitochondrial damage are key pathogenic steps in the development of AP, irrespective of the etiology. The release of endoplasmic reticulum calcium stores by toxins or increased intrapancreatic pressure activates store-operated calcium entry, allowing extracellular calcium to enter the cell through the Orai1 calcium channel. Elevated levels of intracellular calcium lead to premature activation of trypsinogen in pancreatic acinar cells, impairing fluid and HCO3- secretion in ductal cells. Increased and unbalanced reactive oxygen species (ROS) production caused by sustained calcium elevation further contributes to cell dysfunction, leading to mitochondrial damage and cell death [5].

It is essential to treat both pancreatitis and hypercalcemia during the treatment of hypercalcemia-induced AP. Hypercalcemia can be treated with fluids, diuretics, and bisphosphonate, which help transfer calcium from the blood to the bones. Surgery might be needed in specific cases of hormone-producing adenomas [6].

In pancreatitis patients with unknown etiologies, hypercalcemia should always be considered as a potential reason for the disorder. With early diagnosis and therapy, complications can be avoided, and the level of mortality can decrease.

In this study, we aimed to assess the occurrence and severity of hypercalcemia-induced AP and compare it with the clinical characteristics of AP caused by other etiological factors.

## 2. Materials and Methods

### 2.1. Patients and Study Design

Patients with AP (*n* = 4076) aged 18 years or older were enrolled in the prospectively collected, international, multicenter AP registry operated by the Hungarian Pancreatic Study Group (HPSG) between August 2012 and May 2024 [7]. Informed consent was obtained from all participants to publish the information in an online open access publication. Patients under the age of 18 were excluded from the study. The inclusion criteria were based on the definition for hypercalcemia, i.e., a serum calcium level above 2.6 mmol/L. Therefore, AP patients with a serum calcium level above 2.6 mmol/L were included (*n* = 83). These patients were divided into two groups: a hypercalcemia-induced AP group, in which no etiology other than hypercalcemia was present, and a mixed etiology group, in which both hypercalcemia and other etiologies were observed. The control group (*n* = 1245) consisted of patients who had AP with a normal serum calcium level. The etiology of acute pancreatitis in the control group was not hypercalcemia, but alcohol consumption, biliary obstruction, hyperlipidemia, or other factors.

We compared laboratory parameters (CRP, amylase, lipase, WBC, and creatinine), local (pancreatic necrosis, pseudocysts, and fluid) and systemic complications (organ failure), as well as the length of hospital and intensive care stay, and mortality rates of AP.

Data quality analysis revealed the availability of the analyzed parameters in the analyzed and total cohorts. Assuming that the available data consisted of a high number of cases, the collected data were of high quality (Table S1).

### 2.2. Statistical Analysis

We used R version 4.3.1 to analyze these packages: MatchIt v4.5.5, lme4 v1.1.35.3, lmerTest v3.1.1, and emmeans v1.10.2. The statistical analysis was done by Z.A.-T.

Propensity score matching was conducted, adjusting for age, sex, diabetes, lipid disorders, and last pancreatic disease. Using the MatchIt package, we selected 15 controls for each case.

Logarithmic transformation was used for the statistical analysis of the laboratory findings (C-reactive protein, amylase, lipase, leukocyte count, and creatinine), and a mixed linear model with the Tukey post hoc test was employed for the numeric findings (age, C-reactive protein, amylase, lipase, leukocyte count, creatinine and length of hospital stay). A mixed binomial logistic regression was used for the qualitative parameters (severity, organ failure, mortality, and intensive care admission). The random factor was the subclass formed during matching in each model, where each subclass contained one case and the corresponding 15 controls.

### 2.3. Definitions

AP was diagnosed with the two-out-of-three rule (abdominal pain suggestive of pancreatitis, serum amylase, lipase level more significant than three times the upper standard value, or characteristic imaging findings) [8]. All patients provided written informed consent to participate.

Serum calcium level was measured from the first available blood sample of the patient after hospital admission. Normal serum calcium levels range from 2.2 mmol/L to 2.6 mmol/L. We defined hypercalcemia as a serum calcium level above 2.6 mmol/L. Hypercalcemic cases were divided into three subgroups on the basis of the elevation of serum calcium: mild (between 2.6 and 3.0 mmol/L), moderate (between 3.0 and 3.5 mmol/L), and severe hypercalcemia (>3.5 mmol/L).

The severity of AP was categorized by the Revised Atlanta classification from 2012: mild (AP does not involve organ failure or local or systemic complications), moderate (AP involves transient, resolving symptoms in the first 48 h, with single or multiple organ failure or local or systemic complications), and severe (AP involves persistent (beyond 48 h) single or multiple organ failure) [8,9].

Using the modified Marshall Scoring System, organ failure is defined as a score of 2 or more for one of the three organ systems (Table 1) [10].

## 3. Results

### 3.1. Demographics

**Figure 1:**
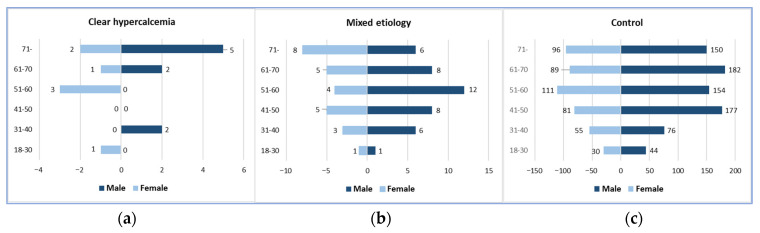
Gender and age distributions: (**a**) Clear hypercalcemia-induced group; (**b**) Mixed etiology group; (**c**) Control group.

A total of 1.20% of our AP patients (16/1328) had a clear hypercalcemic etiology, and 5.05% (67/1328) had a mixed etiology, with 1245 patients in the control group. The mean age of the patients and gender distribution were not significantly different among the three groups. The mean age was 63.31 ± 17.78 years (9 males and 7 females) in the clear hypercalcemic group, 56.66 ± 14.76 years (41 males and 26 females) in the mixed etiology group, and 56.22 ± 15.58 years (783 males and 462 females) in the control group (Figure 1).

**Figure 2:**
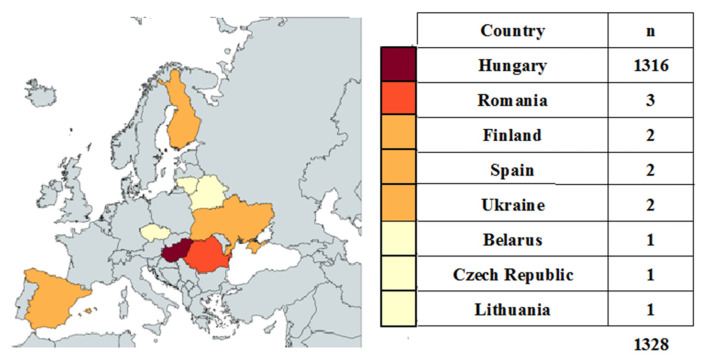
Participating countries.

Map of the participating countries in the study. The map shows the number of patients by country. Created by MapChart (https://www.mapchart.net/europe.html accessed on 5 September 2025) (Figure 2).

### 3.2. Severity of AP

**Figure 3:**
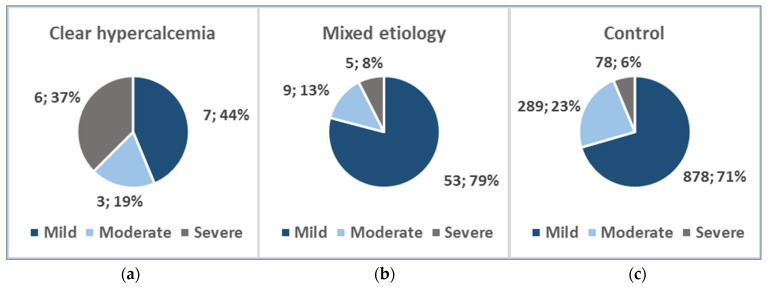
AP severity distribution: (**a**) Clear hypercalcemia-induced group; (**b**) Mixed etiology group; (**c**) Control group.

In the clear hypercalcemia-induced group, there were 6 (37.5%) severe, 3 (18.75%) moderate, and 7 (43.75%) mild AP cases. The mixed etiology group consisted of 5 (7.46%) severe, 9 (13.43%) moderate, and 53 (79.10%) mild AP patients, whereas the control group included 78 (6.27%) severe, 289 (23.21%) moderate, and 878 (70.52%) mild AP patients (Figure 3).

**Figure 4:**
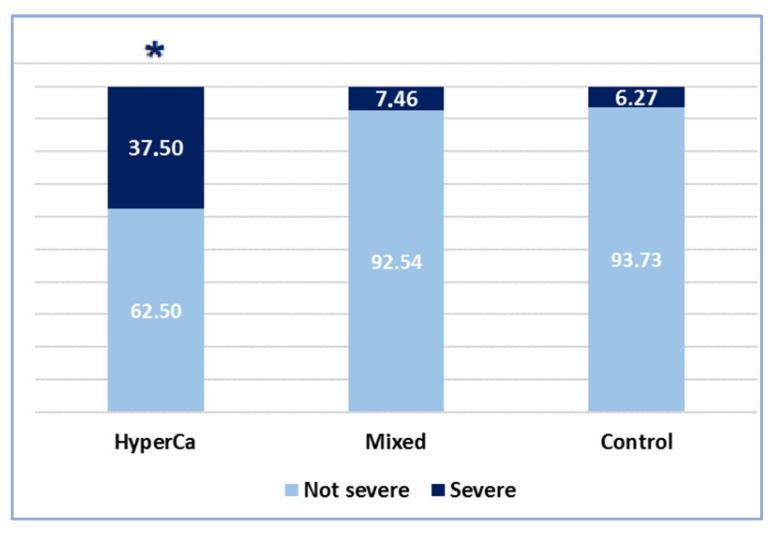
Severity of AP cases (* significant finding).

Severe AP was significantly more common in patients with hypercalcemia-induced AP than in the control group, i.e., 37.5% (6/16) vs. 6.27% (78/1245); odds ratio: 8.98 (95% CI: 2.46–32.68); *p* < 0.001. Severe AP was also significantly more common in the hypercalcemia-induced group than in the mixed etiology group: 37.5% (6/16) vs. 7.46% (5/67); odds ratio: 7.30 (95% CI: 1.36–39.24); *p* = 0.016 (Figure 4).

### 3.3. Complications of AP

Organ failure was more common in the hypercalcemia-induced AP group than in the mixed etiology group, i.e., 55.56% (5/9) vs. 5.77% (3/52); odds ratio: 19.48 (95% CI: 2.25–168.51); *p* = 0.004, and also compared to the control group, i.e., 55.56% (5/9) vs. 11.41% (142/1245); odds ratio: 9.47 (95% CI: 1.81–49.51); *p* = 0.004 (Figure 4).

Heart failure was significantly more common in the clear hypercalcemic group than in the control group: 22.22% (2/9) vs. 3.29% (41/1245); odds ratio: 8.39 (95% CI: 1.24–57.14); *p* = 0.025. There was no significant difference between the clear hypercalcemic and mixed etiology groups: 22.22% (2/9) vs. 3.85% (2/52); odds ratio: 7.14 (95% CI: 0.57–89.43) (Figure 4).

Respiratory failure was significantly more common in the clear hypercalcemic group than in the mixed etiology group: 33.33% (3/9) vs. 3.85% (2/52); odds ratio: 11.54 (95% CI: 1.04–128.04; *p* = 0.045 (Figure 4). We found no significant difference between the clear hypercalcemic and control groups: 33.33% (3/9) vs. 8.76% (109/1245); odds ratio: 4.88 (95% CI: 1.15–27.32).

**Figure 5:**
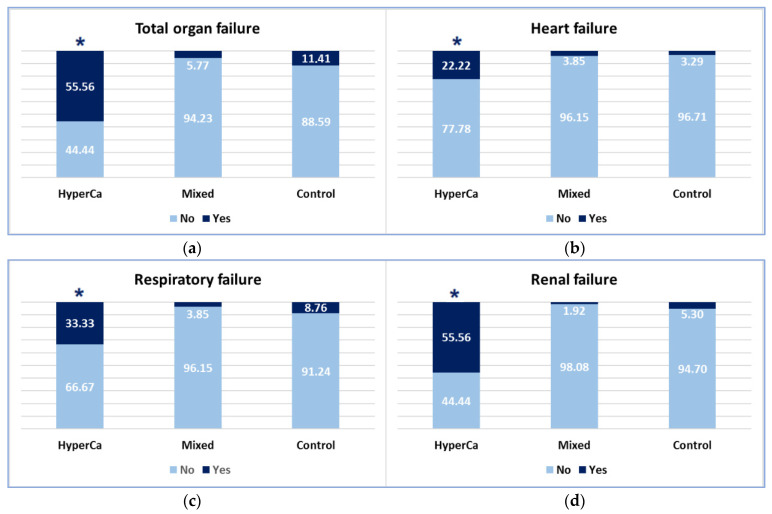
Occurrence of organ failure: (**a**) Total organ failure; (**b**) Heart failure; (**c**) Respiratory failure; (**d**) Renal failure (* significant finding).

Renal failure was more frequent in the hypercalcemia-induced AP group compared to the mixed etiology group, i.e., 55.56% (5/9) vs. 1.92% (1/52); odds ratio: 63.75 (95% CI: 3.72–1092.48); *p* = 0.002, and compared to the control group: 55.56% (5/9) vs. 5.30% (66/1245); odds ratio: 22.32 (95% CI: 4.51–111.11); *p* < 0.001 (Figure 5).

### 3.4. Laboratory Findings

**Figure 6:**
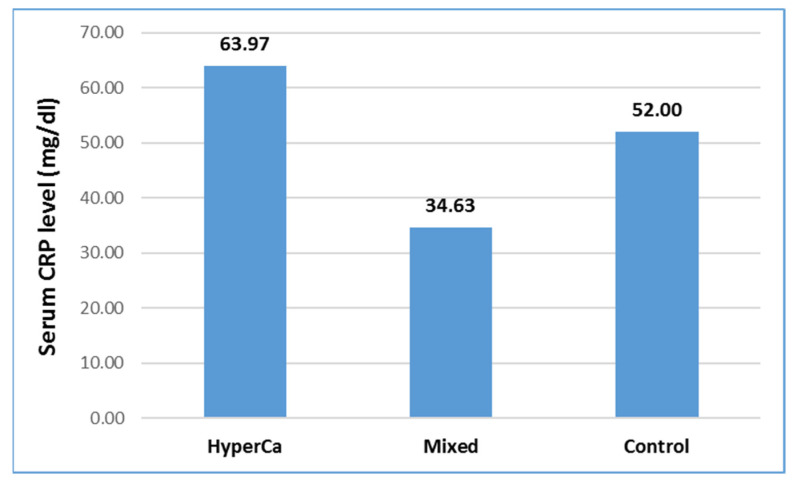
Serum CRP level at admission in acute pancreatitis with different etiologies.

Serum CRP level was not significantly different among the three groups, i.e., control vs. mixed etiology vs. clear hypercalcemia group: 52.0 ± 79.43 vs. 34.63 ± 49.52 vs. 63.97 ± 79.32 mmol/L; control–mixed (97.5% CI: −0.3215–0.5837), *p* = 0.7753; control-clear (97.5% CI: −1.2659–0.6826), *p* = 0.7621; and clear-mixed (97.5% CI: −0.6455–1.4910), *p* = 0.6222 (Figure 6).

**Figure 7:**
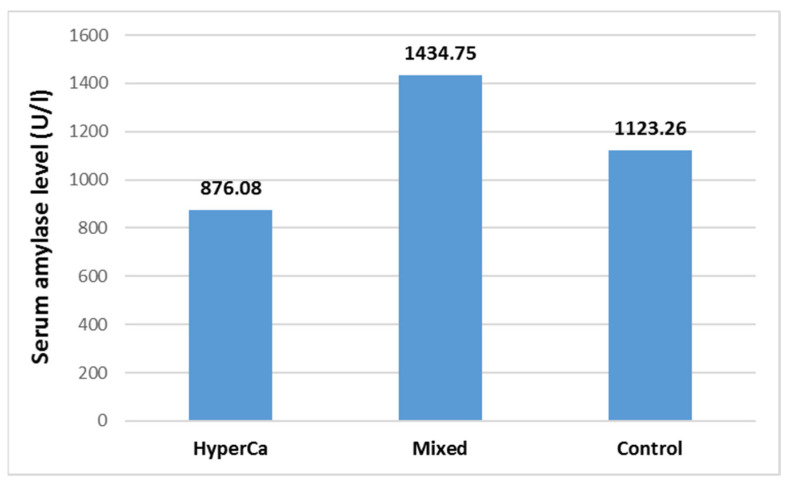
Serum amylase level at admission in acute pancreatitis with different etiologies.

Serum amylase level was not significantly different among the three groups, i.e., control vs. mixed etiology vs. clear hypercalcemia group: 1123.26 ± 1188.32 vs. 1434.75 ± 2106.59 vs. 876.08 ± 807.03 U/l; control–mixed (97.5% CI: −0.5621–0.1746), *p* = 0.4332; control–clear (97.5% CI: −0.5264–1.0033), *p* = 0.7447; and clear–mixed (97.5% CI: −1.2774–0.4129), *p* = 0.4533 (Figure 7).

**Figure 8:**
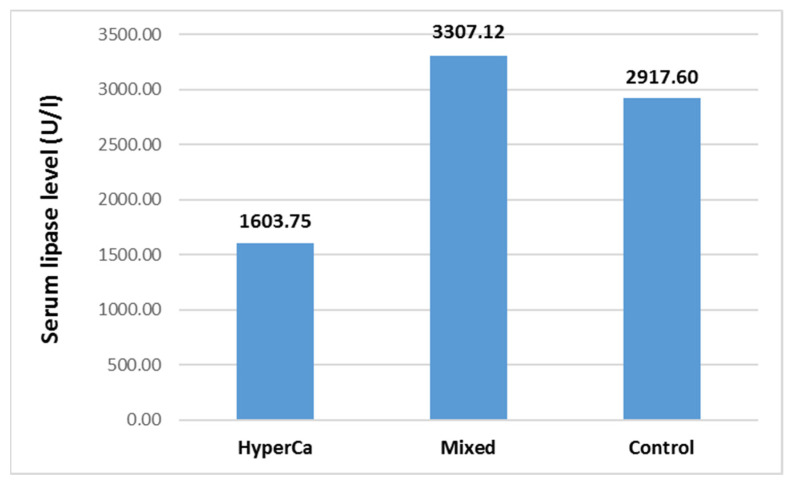
Serum lipase level at admission in acute pancreatitis with different etiologies.

Serum lipase level was not significantly different among the three groups, i.e., control vs. mixed etiology vs. clear hypercalcemia group: 2917.60 ± 3873.94 vs. 3307.12 ± 5460.48 vs. 1603.75 ± 1640.61 U/l; control–mixed (97.5% CI: −0.5585–0.3134), *p* = 0.7869; control–clear (97.5% CI −0.2196–1.5591), *p* = 0.1811; and clear–mixed (97.5% CI: −1.7774–0.1928), *p* = 0.1427 (Figure 8).

**Figure 9:**
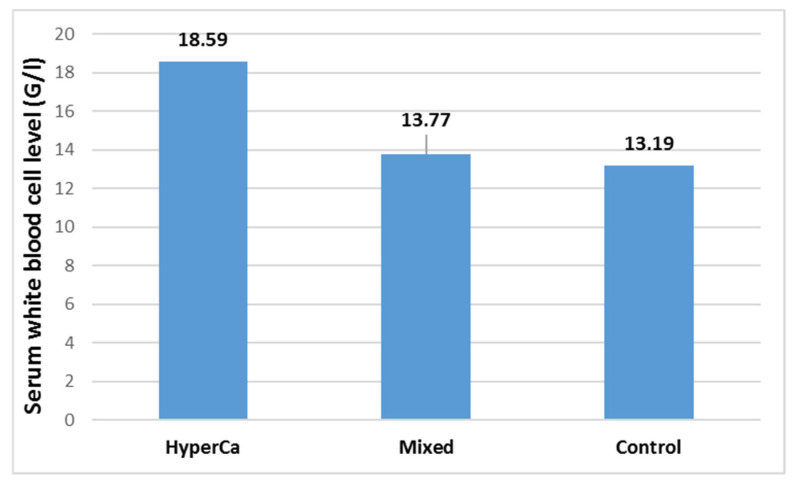
Serum white blood cell levels at admission in acute pancreatitis with different etiologies.

Serum white blood cell level was not significantly different among the three groups, i.e., control vs. mixed etiology vs. clear hypercalcemia group: 13.19 ± 5.29 vs. 13.77 ± 5.36 vs. 18.59 ± 13.46 G/l; control–mixed (97.5% CI: −0.1819–0.0704), *p* = 0.5539; control–clear (97.5% CI −0.4814–0.0364), *p* = 0.1086; and clear–mixed (97.5% CI: −0.1196–0.4531), *p* = 0.3589 (Figure 9).

### 3.5. Severity of Hypercalcemia

Hypercalcemic cases were divided into three subgroups on the basis of the level of serum calcium: mild (between 2.6 and 3.0 mmol/L), moderate (between 3.0 and 3.5 mmol/L), and severe hypercalcemia (>3.5 mmol/L). In the clear hypercalcemic etiology group, the serum white blood cell count was significantly greater in the moderate hypercalcemia group compared to the mild hypercalcemia group: 9.53 ± 3.21 vs. 26.25 ± 17.86, moderate–mild (97.5% CI: 0.12–1.72), *p* = 0.0257. There was no significant difference between the severe–mild hypercalcemia group and the severe–moderate hypercalcemia group: mild vs. moderate vs. severe hypercalcemia group = 9.53 ± 3.21 vs. 26.25 ± 17.86 vs. 20.94 ± 8.27 G/l; severe–mild hypercalcemia group (97.5% CI: −0.14–1.71), *p* = 0.0995; and severe–moderate hypercalcemia group (97.5% CI: −1.06–0.79), *p* = 0.9123.

There were no significant differences in other laboratory parameters (amylase, lipase, leukocyte count, or creatinine) among the three groups according to hypercalcemia grade, nor were there significant differences in mortality, severity of AP, length of hospital stay, or intensive care admission between the groups. The outcome of AP, therefore, was not associated with the severity of hypercalcemia within the hypercalcemic group.

## 4. Discussion

In the present study, we found that 6.25% (83/1328) of AP patients had elevated serum calcium levels (>2.6 mmol/L) and that 16 of them (1.2% of all patients) had no etiology of AP other than hypercalcemia. Hypercalcemia-induced AP is more severe than other etiologies, e.g., renal and cardiac failure are more common.

To the best of our knowledge, to date, there have only been two studies on the severity of hypercalcemia-induced AP, and one of them has only been presented as an abstract [2,11]. Case reports are more common, with approximately 190 patients in 105 case studies [2,12,13,14,15,16,17,18]. Our study comprises the largest number of patients from a rigorously maintained database, providing sufficient power to identify differences in outcomes in this rare cause of AP. Furthermore, we compared laboratory parameters, local (pancreatic necrosis, pseudocysts, and fluid) and systemic (organ failure) complications, hospital and intensive care stay length, and mortality among the different AP etiologies.

The occurrence rates of hypercalcemic AP in previous studies were 3% and 0.83%, respectively [2,11]. This number was 6.25% (83/1328) in our study. Geographical differences and the different distributions of AP etiologies on other continents might explain the greater occurrence in our study.

Compared with patients without hypercalcemia-related pancreatitis, patients with hypercalcemia had an almost twofold greater chance of a lethal outcome during hospitalization (adjusted odds ratio 1.89, 95% CI: 1.31–2.71) [11]. The other study consisted of 100 AP patients, only 3 of which had hypercalcemia as the etiology of AP. The mortality rate among those hypercalcemia-induced AP patients was 0% [2]. In our study, severe pancreatitis together with heart and kidney failure were more frequent in the hypercalcemic group than in the control group; however, mortality did not differ between these two groups. Compared with the findings of the previous study, this difference might be explained by the fact that early diagnosis and optimal therapy among our hypercalcemic AP patients prevented increased mortality.

Consistent with our results, many previous studies have demonstrated that hypercalcemia can cause kidney failure [19,20,21]. Renal insufficiency caused by hypercalcemia can arise via several mechanisms, such as prerenal involvement, alterations in intravascular tone, and changes in glomerular permeability. High blood calcium induces constriction of renal arterioles, although the direct vasoconstrictive effects of excess calcium ions on arteriolar smooth muscle result in a subsequent decline in the glomerular filtration rate and calcium excretion. Furthermore, hypercalcemia may exacerbate the tubular necrosis frequently observed in cases of acute renal failure [19,21]. Another study also demonstrated that hypercalcemia can cause renal failure through diabetes insipidus. Experimental evidence indicates that hypercalcemia inhibits the binding of antidiuretic hormone (ADH) to its receptor and reduces medullary adenylate cyclase activity, a crucial step in the ADH mechanism. Hypercalcemia also inhibits sodium chloride transport out of the ascending limb of Henle, thereby reducing the medullary interstitial sodium content and hypertonicity [21]. This effect could also blunt ADH activity, as well increase the production of medullary prostaglandin E (PGE), a physiologic antagonist of intrarenal ADH activity [22]. A study has shown that hypercalcemia can increase the synthesis of PGE. Chronic hypercalcemia can also lead to nephrocalcinosis and chronic renal insufficiency [23].

Case reports have also shown that hypercalcemia causes acute respiratory failure in the form of acute respiratory distress syndrome through pulmonary calcification and alveolar edema [24,25,26]. Esquivel-Ruiz et al. reported that calcium deposits in the alveolar/capillary barrier can lead to lung injury [27]. Additionally, an experimental study in rats showed that hypercalcemia can increase plasma nitrate, proinflammatory cytokines, and procalcitonin levels via inducible nitric oxide synthase activity, thereby causing sepsis-like syndrome [28].

Calcium homeostasis can also affect cardiac tissue, whereas hypercalcemia can cause shortened QT intervals, prolonged PR intervals, and widened QRS complexes. An experimental study on guinea pigs revealed that hypercalcemia decreases the inward Na/Ca exchange current, which is responsible for the shortening of the action potential [29]. Cardiac manifestations can also include bradycardia, atrioventricular block, and other arrhythmias, which can be life-threatening [30].

The relationship between hypercalcemia and AP is still not fully known today. It has been shown that hypercalcemia leads to increased precipitation of calcium salts in pancreatic juice and accelerates the conversion of trypsinogen to trypsin, which is responsible for the destruction of the pancreatic parenchyma and ducts [31,32]. Hypercalcemia causes inspissation of protein plugs in the pancreatic ducts through the formation of pancreatic calculi [33]. In the case of hypercalcemia caused by hyperparathyroidism, it is postulated that parathyroid hormone plays a role in the pathogenesis of pancreatitis by inhibiting pancreatic vascularisation or by causing the formation of microthrombi, leading to necrosis of the pancreatic parenchyma [34,35]. Although the mechanism is not entirely known, all of these theories could explain why AP caused by hypercalcemia is more severe than AP caused by other etiological factors.

## 5. Conclusions

Our study confirmed that patients with hypercalcemia-induced AP are at a greater risk of developing severe AP and organ failure (renal and cardiac) than patients with other etiologies. The outcome of AP was not associated with the grade of hypercalcemia within the hypercalcemic group. Measuring serum calcium in APs is crucial, because treatment for hypercalcemia should start as early as possible.

## 6. Strengths and Limitations

Our study contains the largest number of hypercalcemia-induced AP patients from a highly controlled database, providing sufficient power to identify differences in outcomes in such a rare form of AP. Although our study consisted of the greatest number of patients ever published, in the case of some parameters, the data were not enough to prove a significant difference due to the small number of patients, resulting from the rarity of the etiology.

## Figures and Tables

**Table 1:** Definitions of organ failure according to the Modified Marshall Organ Failure Score. Scores for patients with preexisting chronic kidney disease depend on the extent of further deterioration of baseline renal function. No formal correction exists for a baseline serum creatinine (≥134 umol/L).

Organ System	Score 0	Score 1	Score 2	Score 3	Score 4
Heart (systolic blood pressure, mm Hg)	>90	<90, fluid responsive	<90, not fluid responsive	<90, pH< 7.3	<90, pH< 7.2
Respiratory (PaO_2_/FiO_2_)	>400	301–400	201–300	101–200	≤101
Renal (serum creatinine, umol/L)	≤134	134–169	170–310	311–439	≥439

## Data Availability

The data presented in this study are available on request from the corresponding author due to other ongoing unpublished studies based on the data from the used register.

## Supplementary Materials

The following supporting information can be downloaded at: https://www.mdpi.com/article/10.3390/jcm14176304/s1.

## Abbreviations

The following abbreviations are used in this manuscript:

ADH	Antidiuretic Hormone
AP	Acute Pancreatitis
CRP	C-Reactive Protein
HPSG	Hungarian Pancreatic Study Group
PGE	Prostaglandin E
ROS	Reactive Oxygen Species
WBC	White Blood Cell
