# Supplementary material for: Hypercalcemia Causes More Severe Acute Pancreatitis: An International Multicenter Cohort Study
Source: J Clin Med. 2025 Sep 6;14(17):6304. doi: 10.3390/jcm14176304 (PMC12429401; doi:10.3390/jcm14176304)
Supplement: Supplementary file 1 [file jcm-14-06304-s001.zip › jcm-3794641-supplementary.pdf]

Supplementary materials

Length of hospital stay was not significantly different between the 3 groups [control vs mixed etiology vs clear hypercalcemia group 8.44± 8.80 vs 8.23± 9.63 vs 9.62± 10.19 days; control–mixed (97.5% CI: -0.01990.3487) p= 0.0917, control-clear (97.5% CI: -0.0436-0.7199) p= 0.0947; clear-mixed (97.5% CI: -0.5954-0.2479) p= 0.5980] (Figure S1).

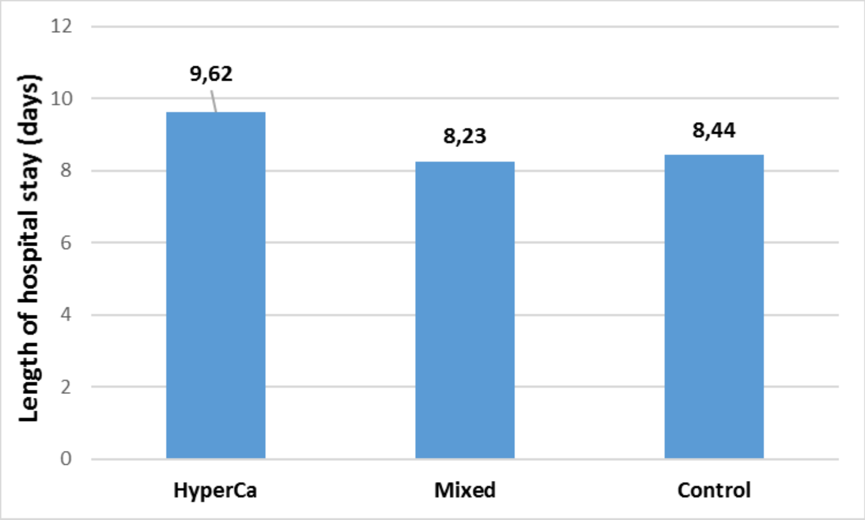

Figure S1. Length of hospital stay in acute pancreatitis with different etiologies.

Mortality was not significantly different between the 3 groups [control vs mixed etiology vs clear hypercalcemia 2.65% (33/1245) vs 0% (0/57) vs 15.38% (2/13); control–mixed (95% CI: 0.0000-Inf) p= 0.9999, control-clear (95% CI: 0.0216-1.2736) p= 0.0970, clear-mixed (95% CI: 0.0000-Inf) p= 0.9999] (Figure S2).

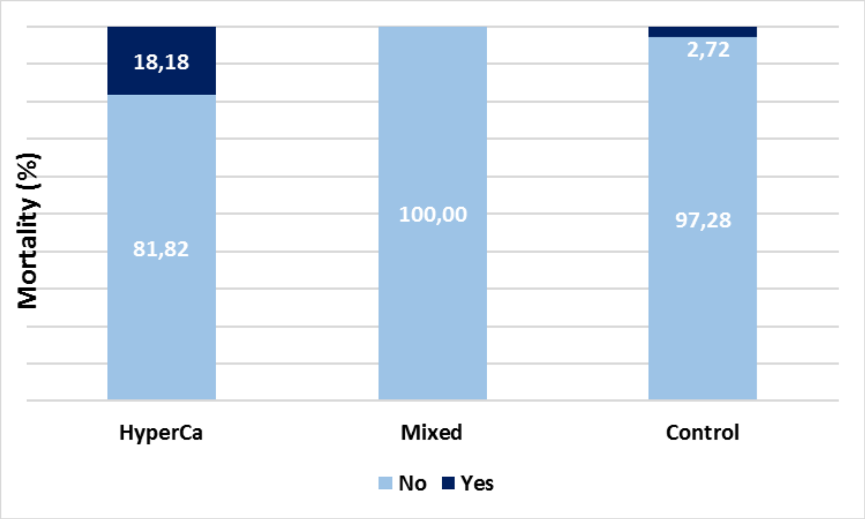

Figure S2. Mortality in acute pancreatitis with different etiologies.

Intensive care admission was not significantly different between the 3 groups [control vs mixed etiology vs clear hypercalcemia 3.91% (47/1202) vs 3.51% (2/57) vs 0% (0/11); control–mixed (95% CI: 0.1998-6.2667) p= 0.9872, control-clear (95% CI: 0.0000-Inf) p= 1.0000, clear-mixed (95% CI: 0.0000-Inf) p= 1.0000] (Figure S3).

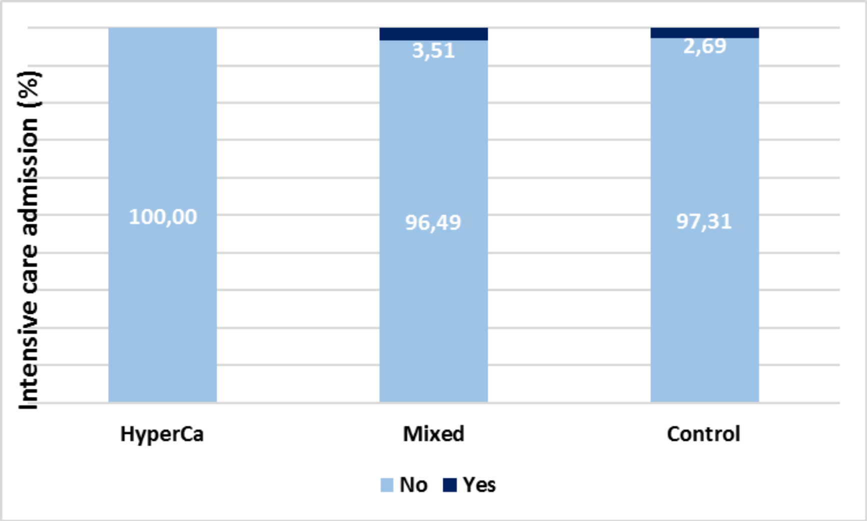

Figure S3. Intensive care admission in acute pancreatitis with different etiologies.

The presence of pancreas necrosis was not significantly different between the 3 groups [control vs mixed etiology vs clear hypercalcemia 2.69% (27/1003) vs 2.00% (1/50) vs 0% (0/11); control–mixed (95% CI: 0.1216-15.1113) p= 0.9530, control-clear (95% CI: 0.0000-Inf) p= 1.0000, clear-mixed (95% CI: 0.0000-Inf) p= 1.0000] (Figure S4).

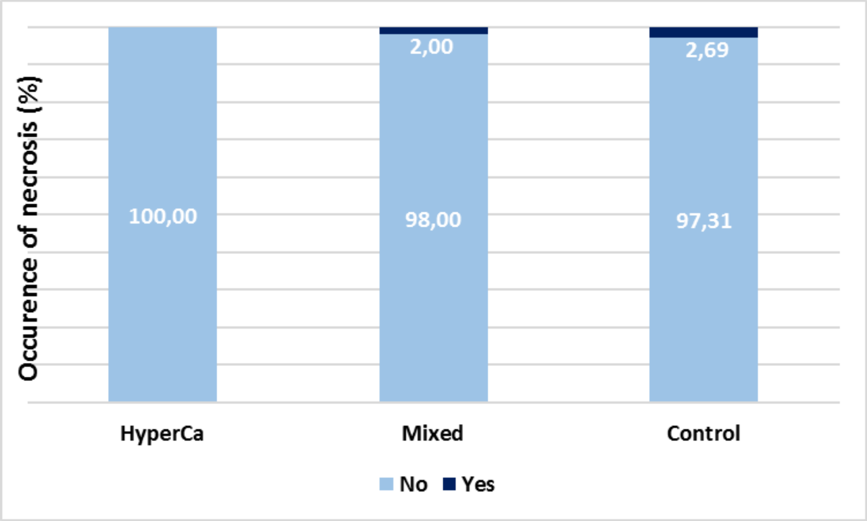

Figure S4. Occurrence of necrosis in acute pancreatitis with different etiologies.

The occurence of pseudocyst was not significantly different between the 3 groups [control vs mixed etiology vs clear hypercalcemia 2.19% (22/1003) vs 2.00% (1/50) vs 9.09% (1/11); control–mixed (95% CI:

0.0976-12.3680) p= 0.9954, control-clear (95% CI: 0.0182-2.7581) p= 0.3429, clear-mixed (95% CI: 0.1614-148.7178) p= 0.5194] (Figure S5).

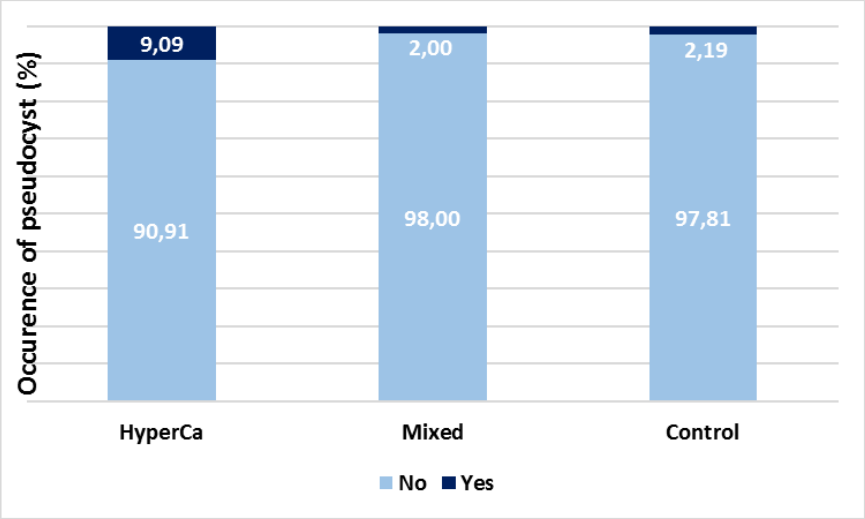

Figure S5. Occurrence of pseudocyst in acute pancreatitis with different etiologies.

Clear hypercalcemia-induced group

In the clear hypercalcemic group there was no significant difference in serum CRP level [mild vs moderate vs severe hypercalcemia group 36.35± 58.69 vs 89.96± 110.68 vs 57.47± 40.74 mmol/l; moderate–mild (97.5% CI: -2.28-4.06) p= 0.7227, severe-mild (97.5% CI: -2.23-4.99) p= 0.5545, severe-moderate (97.5% CI: -2.96-3.95) p= 0.9160] (Figure S6).

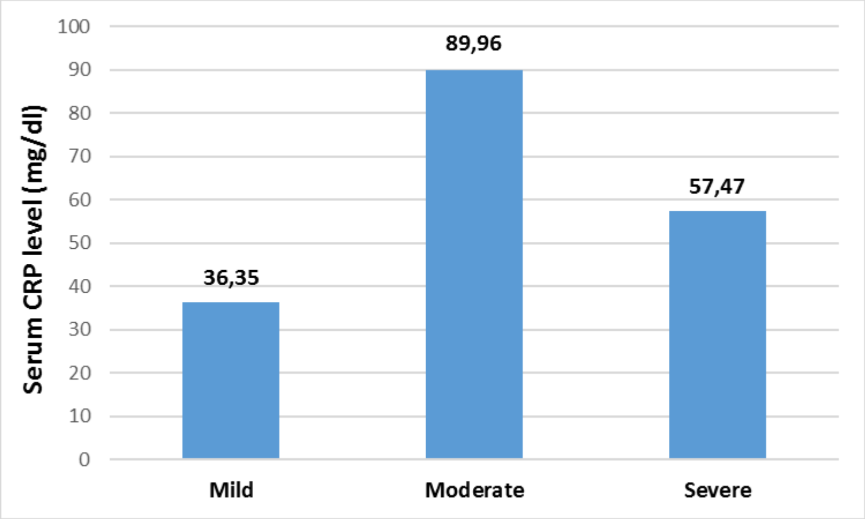

Figure S6. Serum CRP level at admission in clear hypercalcemia-induced acute pancreatitis with different severity of hypercalcemia.

Serum amylase level was not significantly different between the 3 groups [mild vs moderate vs severe hypercalcemia group 604.00± 616.13 vs 664.80± 492.07 vs 1681.67± 1180.73 U/l; moderate–mild (97.5% CI: -1.31-2.49) p= 0.6833, severe-mild (97.5% CI: -0.67-3.73) p= 0.1858; severe-moderate (97.5% CI: -1.25-3.14) p= 0.4924] (Figure S7).

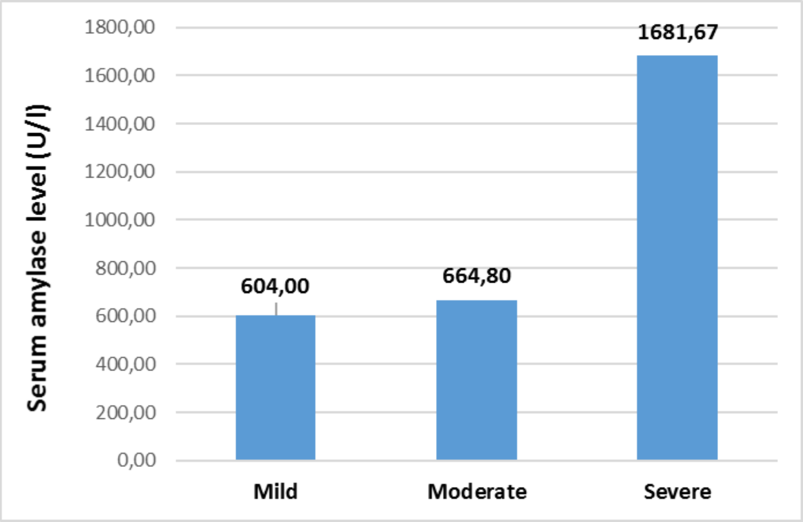

Figure S7. Serum amylase level at admission in clear hypercalcemia-induced acute pancreatitis with different severity of hypercalcemia.

Serum lipase level was not significantly different between the 3 groups [mild vs moderate vs severe hypercalcemia group 1433.50± 2403.91 vs 1766.00± 1237.98 vs 1560.33± 1715.80 U/l; moderate–mild (97.5% CI: -1.38-4.07) p= 0.3908, severe-mild (97.5% CI: -2.07-4.14) p= 0.6347, severe-moderate (97.5% CI: -3.28-2.66) p= 0.9544] (Figure S8).

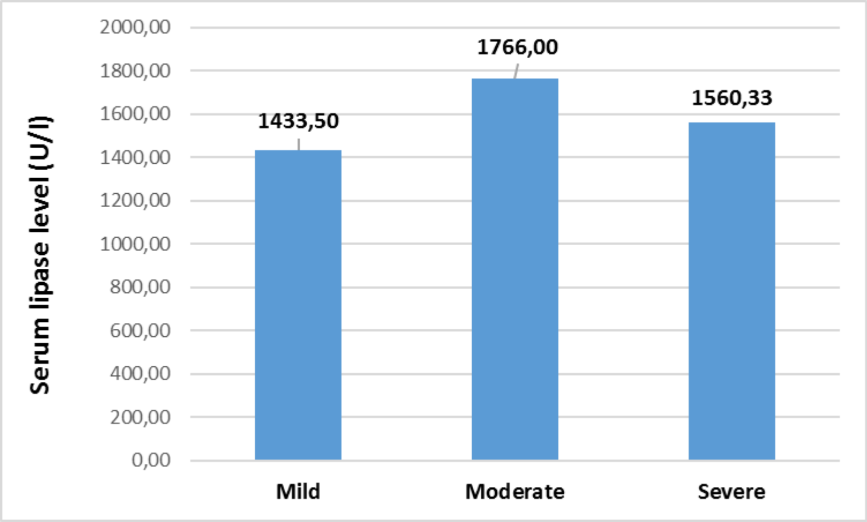

Figure S8. Serum lipase level at admission in clear hypercalcemia-induced acute pancreatitis with different severity of hypercalcemia.

Length of hospital stay was not significantly different between the 3 groups [mild vs moderate vs severe hypercalcemia group 13.00± 10.44 vs 9.40± 11.78 vs 4.33± 7.51 days; moderate–mild (97.5% CI: -2.66-1.96) p= 0.9115, severe-mild (97.5% CI: -3.96-1.38) p= 0.4133, severe-moderate (97.5% CI: -3.61-1.72) p= 0.6113] (Figure S9).

severe-mild (95% CI: 0.0000-Inf) p= 1.0000, severe-moderate (95% CI: 0.0181-13.7984) p= 0.8706] (Figure S11).

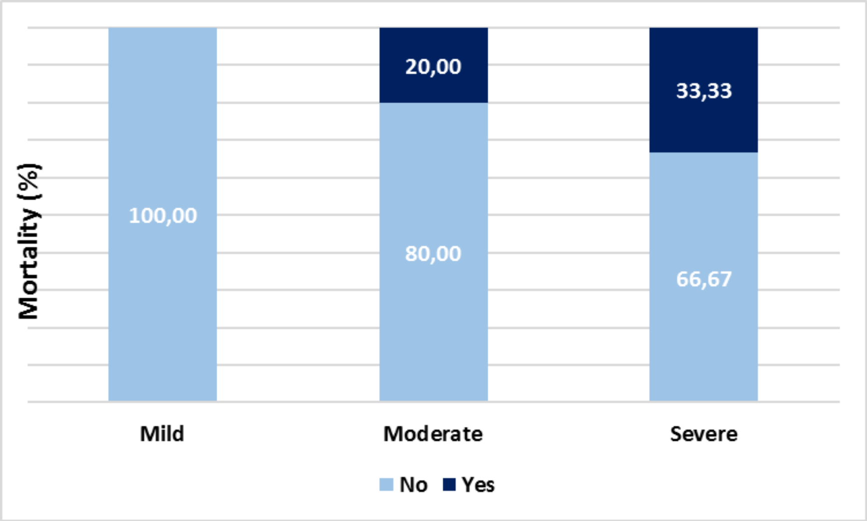

Figure S11. Mortality in clear hypercalcemia-induced acute pancreatitis with different severity of hypercalcemia.

The occurrence of organ failure was not significantly different between the 3 groups [mild vs moderate vs severe hypercalcemia 25% (1/4) vs 75% (3/4) vs 100% (1/1); moderate–mild (95% CI: 0.0016-7.5413) p= 0.4283, severe-mild (95% CI: 0.0000-Inf) p= 1.0000, severe-moderate (95% CI: 0.0000-Inf) p= 1.0000] (Figure S12).

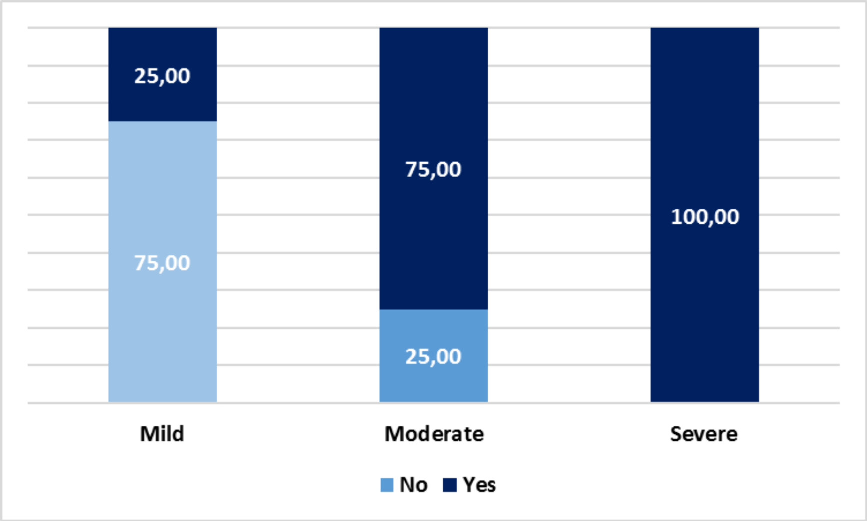

Figure S12. Occurrence of organ failure in clear hypercalcemia-induced acute pancreatitis with different severity of hypercalcemia.

Mixed etiology-induced group

In the mixed etiology group there was no significant difference in serum CRP level [mild vs moderate vs severe hypercalcemia group 31.66± 51.69 vs 75.77± 48.59 vs 36.32± 31.35 mmol/l; moderate–mild (97.5% CI: -0.34-3.66) p= 0.1218, severe-mild (97.5% CI: -0.61-1.97) p= 0.4149, severe-moderate (97.5% CI: -3.25-1.29) p= 0.5558] (Figure S13).

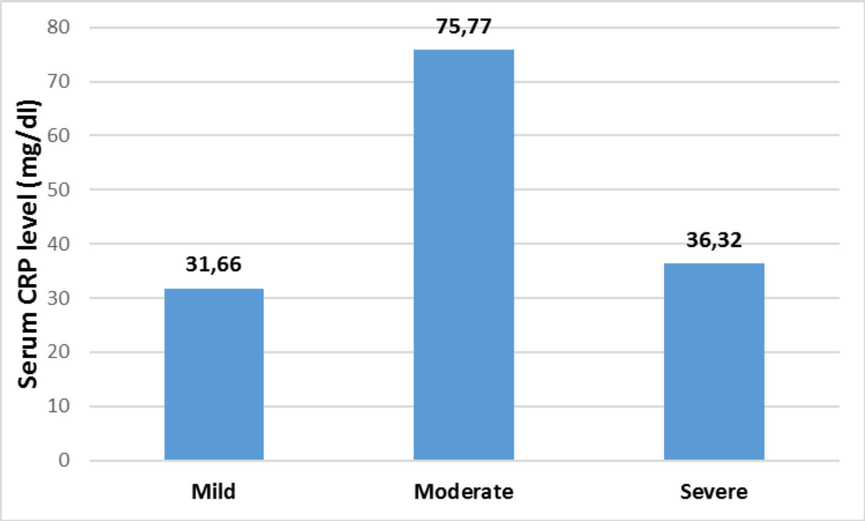

Figure S13. Serum CRP level at admission in mixed etiology-induced acute pancreatitis with different severity of hypercalcemia.

Serum amylase level was not significantly different between the 3 groups [mild vs moderate vs severe hypercalcemia group 1437.46± 2314.80 vs 1236.33± 985.22 vs 1493.62± 870.32 U/l; moderate–mild (97.5% CI: -1.63-1.75) p= 0.9961, severe-mild (97.5% CI: -0.53-1.64) p= 0.4426, severe-moderate (97.5% CI: -1.43-2.42) p= 0.8095] (Figure S14).

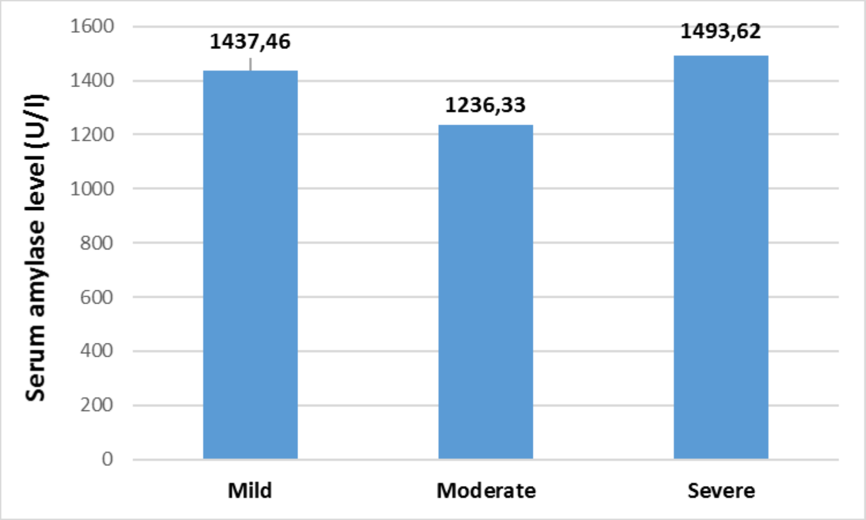

Figure S14. Serum amylase level at admission in mixed etiology-induced acute pancreatitis with different severity of hypercalcemia.

Serum lipase level was not significantly different between the 3 groups [mild vs moderate vs severe hypercalcemia group 3545.74± 5929.43 vs 1759.67± 1004.25 vs 2410.50± 2566.77 U/l; moderate–mild (97.5% CI: -1.92-1.81) p= 0.9970, severe-mild (97.5% CI: -1.29-1.44) p= 0.9900; severe-moderate (97.5% CI: -2.07-2.34) p= 0.9883] (Figure S15).

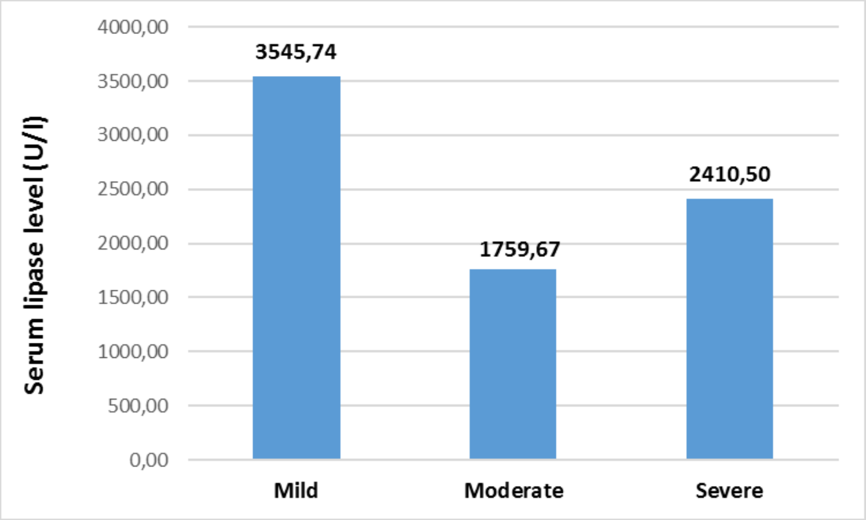

Figure S15. Serum lipase level at admission in mixed etiology-induced acute pancreatitis with different severity of hypercalcemia.

Serum white blood cell level was not significantly different between the 3 groups [mild vs moderate vs severe hypercalcemia group 13.52± 5.10 vs 13.70± 4.72 vs 15.42± 7.59 G/l; moderate–mild (97.5% CI: -0.52-0.58) p= 0.9891, severe-mild (97.5% CI: -0.27-0.48) p= 0.7702, severe-moderate (97.5% CI: -0.56-0.71) p= 0.9566] (Figure S16).

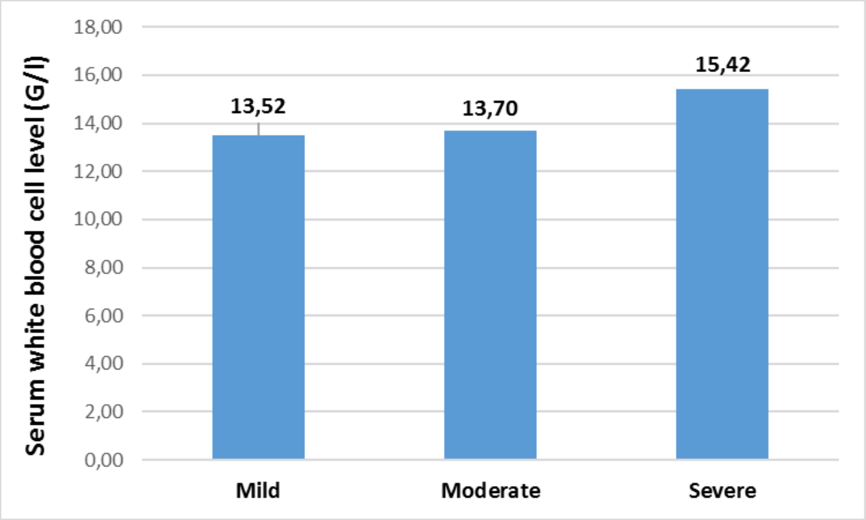

Figure S16. Serum white blood cell level at admission in mixed etiology-induced acute pancreatitis with different severity of hypercalcemia.

Length of hospital stay was not significantly different between the 3 groups [mild vs moderate vs severe hypercalcemia group 6.93± 7.41 vs 20.00± 21.66 vs 11.25 ± 13.19 days; moderate–mild (97.5% CI: -0.81-1.56) p= 0.7286, severe-mild (97.5% CI: -0.69-0.84) p= 0.9714, severe-moderate (97.5% CI: -1.65-1.05) p= 0.8522] (Figure S17).

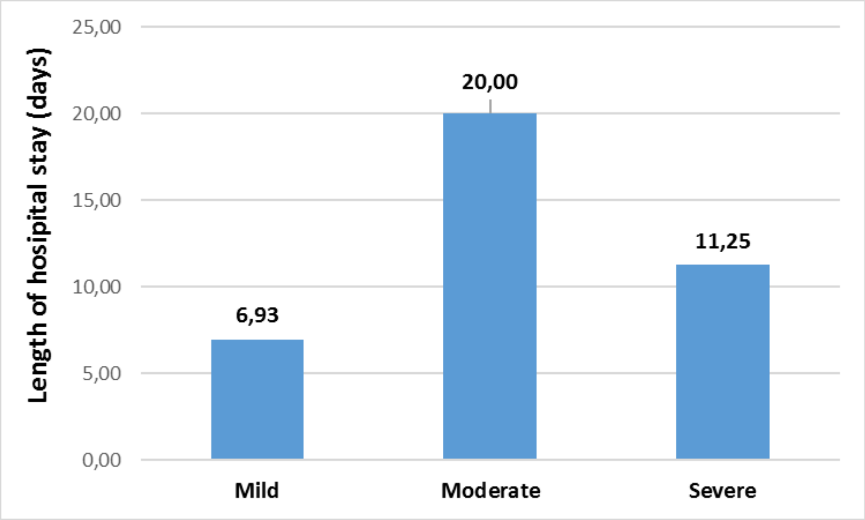

Figure S17. Length of hospital stay in mixed etiology-induced acute pancreatitis with different severity of hypercalcemia.

The occurrence of severe AP was not significantly different between the 3 groups [mild vs moderate vs severe hypercalcemia 7.27% (4/55) vs 0.00% (0/3) vs 11.11% (1/9); moderate–mild (97.5% CI: 0.0000-Inf) p= 1.0000, severe-mild (97.5% CI: 0.0447-8.8052) p= 0.9058, severe-moderate (97.5% CI: 0.0000-Inf) p= 1.0000] (Figure S18).

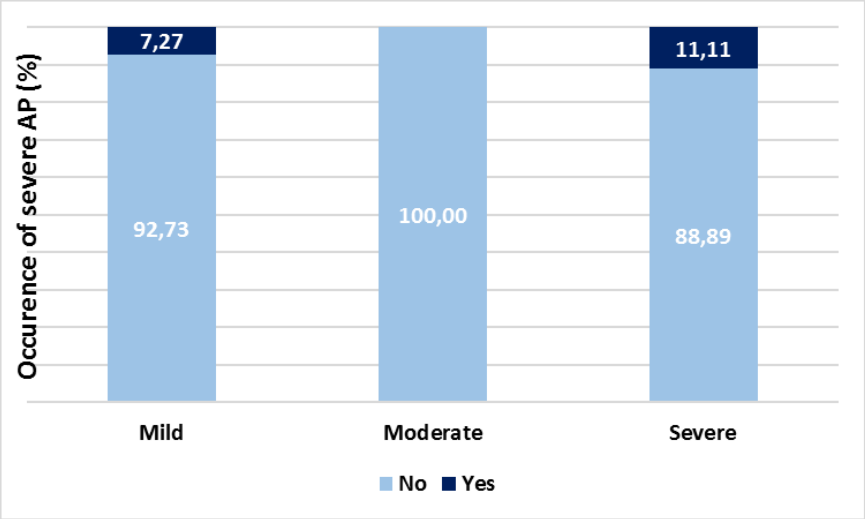

Figure S18. Severity of mixed etiology-induced acute pancreatitis with different severity of hypercalcemia.

Mortality was not significantly different between the 3 groups [mild vs moderate vs severe hypercalcemia 0% (0/46) vs 0% (0/3) vs 0% (0/8).

The occurrence of organ failure was not significantly different between the 3 groups [mild vs moderate vs severe hypercalcemia 4.55% (2/44) vs 0.00% (0/2) vs 16.67% (1/6); moderate–mild (97.5% CI: 0.0000--Inf) p= 1.0000, severe-mild (97.5% CI: 0.0123-4.6247) p= 0.4805, severe-moderate (97.5% CI: 0.0000-Inf) p= 1.0000] (Figure S19).

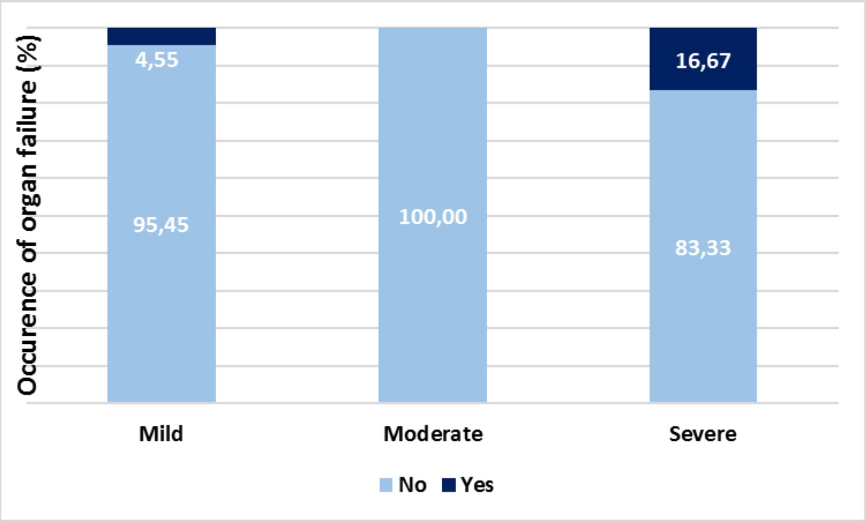

Figure S19. Occurrence of organ failure in mixed etiology-induced acute pancreatitis with different severity of hypercalcemia.

Data availability (%)	Clear etiology	Mixed etiology	Control	Analyzed cohort	Total cohort
Sex	100	100	100	100	100
Age	100	100	100	100	100
AP etiology	100	100	100	100	100
Severity	100	100	100	100	100
Mortality	81.3	85.1	100	99.0	99.7
Previous pancreas disease	100	100	100	100	
Organ failure	75.0	85.1	97.8	98.3	
Amylase	81.3	85.1	92.6	92.1	
Lipase	75.0	76.1	82.3	81.9	
White Blood Cell number	81.3	83.6	95.4	94.7	
Creatinine	81.3	83.6	92.2	91.6	
C reactive Protein	75.0	85.1	91.8	91.3	
Intervention	75.0	85.1	99.5	98.5	
Intensive therapy	68.8	85.1	96.5	95.6	
Pancreatic complication	68.8	74.6	80.6	80.1	

Table S1. Data quality analysis.

Supplementary figure legends

Figure S1. Length of hospital stay in acute pancreatitis with different etiologies.

Figure S2. Mortality in acute pancreatitis with different etiologies.

Figure S3. Intensive care admission in acute pancreatitis with different etiologies.

Figure S4. Occurrence of necrosis in acute pancreatitis with different etiologies.

Figure S5. Occurrence of pseudocyst in acute pancreatitis with different etiologies.

Figure S6. Serum CRP level at admission in clear hypercalcemia-induced acute pancreatitis with different severity of hypercalcemia.

Figure S7. Serum amylase level at admission in clear hypercalcemia-induced acute pancreatitis with different severity of hypercalcemia.

Figure S8. Serum lipase level at admission in clear hypercalcemia-induced acute pancreatitis with different severity of hypercalcemia.

Figure S9. Length of hospital stay in clear hypercalcemia-induced acute pancreatitis with different severity of hypercalcemia.

Figure S10. Severity of acute pancreatitis in clear hypercalcemia-induced acute pancreatitis with different etiology of hypercalcemia.

Figure S11. Mortality in clear hypercalcemia-induced acute pancreatitis with different severity of hypercalcemia.

Figure S12. Occurrence of organ failure in clear hypercalcemia-induced acute pancreatitis with different severity of hypercalcemia.

Figure S13. Serum CRP level at admission in mixed etiology-induced acute pancreatitis with different severity of hypercalcemia.

Figure S14. Serum amylase level at admission in mixed etiology-induced acute pancreatitis with different severity of hypercalcemia.

Figure S15. Serum lipase level at admission in mixed etiology-induced acute pancreatitis with different severity of hypercalcemia.

Figure S16. Serum white blood cell level at admission in mixed etiology-induced acute pancreatitis with different severity of hypercalcemia.

Figure S17. Length of hospital stay in mixed etiology-induced acute pancreatitis with different severity of hypercalcemia.

Figure S18. Severity of mixed etiology-induced acute pancreatitis with different severity of hypercalcemia.

Figure S19. Occurrence of organ failure in mixed etiology-induced acute pancreatitis with different severity of hypercalcemia.
